# Phospholipase C-ε Regulates Epidermal Morphogenesis in *Caenorhabditis elegans*

**DOI:** 10.1371/journal.pgen.1000043

**Published:** 2008-03-28

**Authors:** Rafael P. Vázquez-Manrique, Anikó I. Nagy, James C. Legg, Olivia A. M. Bales, Sung Ly, Howard A. Baylis

**Affiliations:** Department of Zoology, University of Cambridge, Cambridge, United Kingdom; Stanford University Medical Center, United States of America

## Abstract

Migration of cells within epithelial sheets is an important feature of embryogenesis and other biological processes. Previous work has demonstrated a role for inositol 1,4,5-trisphosphate (IP_3_)-mediated calcium signalling in the rearrangement of epidermal cells (also known as hypodermal cells) during embryonic morphogenesis in *Caenorhabditis elegans*. However the mechanism by which IP_3_ production is stimulated is unknown. IP_3_ is produced by the action of phospholipase C (PLC). We therefore surveyed the PLC family of *C. elegans* using RNAi and mutant strains, and found that depletion of PLC-1/PLC-ε produced substantial embryonic lethality. We used the epithelial cell marker *ajm-1::gfp* to follow the behaviour of epidermal cells and found that 96% of the arrested embryos have morphogenetic defects. These defects include defective ventral enclosure and aberrant dorsal intercalation. Using time-lapse confocal microscopy we show that the migration of the ventral epidermal cells, especially of the leading cells, is slower and often fails in *plc-1(tm753)* embryos. As a consequence *plc-1* loss of function results in ruptured embryos with a Gex phenotype (gut on exterior) and lumpy larvae. Thus PLC-1 is involved in the regulation of morphogenesis. Genetic studies using gain- and loss-of-function alleles of *itr-1*, the gene encoding the IP_3_ receptor in *C. elegans*, demonstrate that PLC-1 acts through ITR-1. Using RNAi and double mutants to deplete the other PLCs in a *plc-1* background, we show that PLC-3/PLC-γ and EGL-8/PLC-β can compensate for reduced PLC-1 activity. Our work places PLC-ε into a pathway controlling epidermal cell migration, thus establishing a novel role for PLC-ε.

## Author Summary

Morphogenesis is a fundamental part of development which underlies the ability of animals, including humans, to define the shape of their tissues and organs and thus enable their proper function. To understand morphogenesis we need to understand the signalling networks that regulate coordinated changes in cell morphology, movement and adhesion. We know that in *C. elegans* intracellular signalling through the messenger inositol 1,4,5-trisphosphate (IP_3_) is required for the proper completion of the morphogenetic processes. However the mechanism by which this signal is produced remains unclear. In this work we define the mechanism responsible for IP_3_ production in *C. elegans*. We use a combination of genetic and morphological analysis to show that phospholipase C-epsilon (PLC-ε) is the molecule responsible for IP_3_ production. In worms with disrupted PLC-ε the embryonic epidermal cells fail to migrate properly so that morphogenesis fails. PLC-ε was only discovered relatively recently and interacts directly with a wide range of signalling pathways, including others that are known to regulate important cellular properties during morphogenesis such as small GTPases. Therefore we establish a potential link between IP_3_ signalling and other pathways that are known to be involved in cell movements. This is an important advance in defining the network of interactions that regulate epithelial cell movements in morphogenesis.

## Introduction

Morphogenesis is a fundamental aspect of animal development, during which organs and tissues are formed. During morphogenesis a programmed series of migrations and fusions of epithelial sheets take place. These are finely coordinated by signalling pathways (review by Bard [1]). The process of wound healing, after tissue damage, recapitulates many of the traits of epithelial morphogenesis, so that understanding morphogenesis is also relevant to this aspect of human health [2],[3]. Dorsal closure in *Drosophila* [4], and ventral enclosure in *C. elegans* [5]–[7], are both paradigmatic models of morphogenesis. Due to its genetic tractability dorsal closure in *Drosophila* has become the best characterised example of epithelial morphogenesis and it is clear that many of its features are shared by other systems [8]. Ventral enclosure in *C. elegans* is another important model which is providing new insights into epithelial morphogenesis [5],[7].

The epidermis (also known as the hypodermis) plays a key role during morphogenesis in *Caenorhabditis elegans*. Just after gastrulation the epidermis is organised in a layer of six rows of cells, located on the dorsal side of the embryo. The subsequent epidermal morphogenesis can be divided into three major events [5]–[7]: (1) dorsal intercalation, during which the two dorsal most rows of cells interdigitate to form a single row of cells; (2) ventral enclosure, during which the ventral-most rows of cells migrate to the ventral side of the embryo and establish junctional connections with symmetric opposing cells and (3) elongation, in which forces generated by circular filaments, located within the epidermal cells, drive the change from an ovoid to a worm-shaped larva. Neuroblasts and body wall muscle precursors provide the substrate for migration of epidermal cells. Many kinds of molecules are involved in the coordination of morphogenesis, including proteins of the cytoskeleton and adherens junction, and cell signalling molecules [7]. Among the later, IP_3_ signalling has recently been shown to play an important role in morphogenesis, by regulating the organisation of the actin cytoskeleton during epidermal cell migration [9].

IP_3_ signalling is a fundamental mechanism by which animal cells transduce extracellular signals into intracellular calcium signals, which in turn regulates a wide range of cellular responses. At the heart of this process is the IP_3_ receptor (IP_3_R), a large channel protein located within the ER membrane, which modulates calcium release in reponse to IP_3_ production (reviewed in [10]–[12]). Despite its importance in signal transduction little is known about either the functions of IP_3_ signalling during embryonic development or of the mechanisms regulating its production in developmental events (reviewed by Whitaker [13]). The IP_3_ receptor is encoded in *C. elegans* by a single gene, *itr-1* [14]. It has been shown that disruption of IP_3_ signalling in *C. elegans* compromises embryonic development [9],[15]. For example, transient disruption of IP_3_ signalling, by means of an IP_3_ “sponge”, produces embryonic arrest [15]. Moreover, the cold sensitive mutant of the IP_3_ receptor, *itr-1(jc5)*, produces up to 95% dead embryos at 15°C, whilst the temperature-sensitive mutant of *itr-1(sa73)* produces around 20% embryonic lethality at 20°C (a partially restrictive temperature) [9]. Both mutants, *jc5* and *sa73*, produce arrested embryos due to defects during morphogenesis. Therefore IP_3_ signalling through ITR-1 is required during *C. elegans* embryonic development, and has a role in regulating morphogenesis. Despite the importance of IP_3_ signalling for appropriate progression of morphogenesis, little is known about the network of molecules that function in this pathway to regulate epidermal cell behaviour.

IP_3_ is produced by hydrolysis of phosphatidylinositol 4,5-bisphosphate (PIP_2_) catalysed by phospholipase C (PLC). To date six isoforms of PLC have been described: PLC-β, PLC-γ, PLC-δ, PLC-ε, PLC-ζ, and PLC-η [16]–[19]. PLCs are modular proteins, which share common motifs but also contain family-specific regulatory domains, making them susceptible to different and complex modes of regulation. The recently discovered isoform, PLC-ε, is an exemplary case of complex regulation among PLCs. PLC-1/PLC-ε was isolated in *C. elegans* as a LET-60/Ras interacting molecule [17]. Mammalian PLC-ε proteins are both effectors and regulators of small GTPases of the Ras and Rho families [20], and are thus able to play a pivotal role between small GTPase and IP_3_-mediated calcium signalling. In *C. elegans* there are five active PLC isozymes belonging to four of the six families: *plc-2* and *egl-8* (PLC-β), *plc-3* (PLC-γ), *plc-4* (PLC-δ), and *plc-1* (PLC-ε) [21]. Both PLC-1 and PLC-3 regulate ovulation [22],[23], and a number of other functions have been described for PLC-3 and EGL-8 [21],[24],[25].

Here we identify PLC-1/PLC-ε as a component of the network of molecules that regulates *C. elegans* morphogenesis. We show that PLC-1 is required for epidermal morphogenesis. PLC-1 depleted embryos have defects in ventral migration and also in dorsal intercalation. As a consequence, *plc-1* loss of function results in ruptured embryos, with a Gex phenotype, and lumpy larvae. We show that two other PLCs, PLC-3/PLC-γ and EGL-8/PLC-β, can compensate for a lack of PLC-1 activity in morphogenesis. We demonstrate that PLC-1 acts through the IP_3_ receptor of *C. elegans* (ITR-1), a molecule known to be involved in the regulation of *C. elegans* morphogenesis. Therefore our results suggest that PLC-1 is a key molecule in a pathway which regulates the cytoskeleton during epidermal migration. Further, the properties of PLC-1 mean that it may be an integrator of IP_3_/Ca^2+^ and small GTPase signalling pathways.

## Results/Discussion

### Identification of Phospholipase C Genes which Regulate Embryonic Development

**Figure 1:**
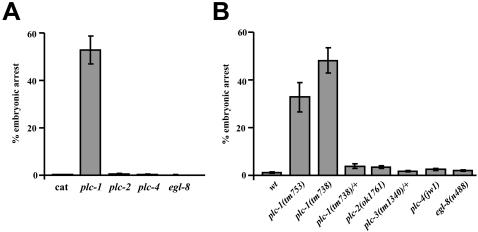
Reduction of *plc-1* results in embryonic arrest. (A) Embryonic arrest resulting from RNAi of *plc* genes in wild type worms. Only RNAi of *plc-1* produces a significant level of embryonic arrest. (B) Embryonic arrest resulting from loss-of-function mutants in the five active *plc* genes. Only *plc-1* mutants have high levels of embryonic arrest. In both histograms, data is shown as the mean percentage lethality from the offspring of 10–12 individual worms. Error bars represent SEMs.

Signalling through inositol 1,4,5-trisphosphate regulates development in *C. elegans*. Therefore, we hypothesised that ablation of PLC function should result in embryonic arrest due to decreased IP_3_ signalling. We tested the function of the five genes encoding active PLCs (*plc-1*, *plc-2*, *plc-3*, *plc-4* and *egl-8*) using both RNAi, and mutant strains (Figure 1). Of the five PLCs, only reduction of PLC-1/PLC-ε function resulted in a clear and substantial increase in embryonic lethality. Thus, *plc-1* activity is required for successful embryonic development. Depletion of *egl-8*, *plc-2* and *plc-4* had no effect on embryonic survival (Figure 1).

Complete depletion of *plc-3* in null mutants, *plc-3(tm1340)*, or by RNAi produces sterility [24], so embryogenesis is not readily observed in these worms. However, some of the *plc-3(RNAi)* animals laid a small number of embryos before becoming sterile, 58% of which arrested (N = 30). *plc-3(tm1340)*/+ hermaphrodites do not have increased lethality (Figure 1, Table 1), but this could result from maternal rescue. In contrast *plc-3(tm1340)* homozygous animals rescued with an unstable extrachromosomal array of the *plc-3* gene, produced a small but significant increase in the number of dead embryos (Table 1). Such an array will be absent from a proportion of zygotes and is unlikely to be expressed in the germline (a general property of *C. elegans* extrachromnosmal arrays). This suggests that PLC-3 may also be required during embryonic development.

**Table 1:** Embryonic arrest caused by PLC genes.

Genotype	% of embryonic arrest	N
Wild type	1.1	2065
*plc-1(tm753)*	32.8	299
*plc-1(tm753)*; *jwEx302[plc-1(+)]* a	0.9	3079
*plc-1(tm753)/+*	3.4	2844
*plc-1(tm738)*	48.1	190
*plc-1(tm738)/+*	4.1	2225
*plc-2(ok1761)*	2.3	1754
*plc-4(jw1)*	2.4	1655
*egl-8(n488)*	3.9	638
*plc-3(tm1340)/+* b	1.8	2058
*plc-3(tm1340)*; *jwEx311[plc-3(+)]* a	8.1	1138
*plc-1(tm753)*; *plc-3(tm1340)/+* b	52.3	348
*plc-1(tm753)*; *plc-3*(*tm1340*); *jwEx311[plc-3(+)]* a	43.5	345
*plc-1(tm753)*; *egl-8(n488)*	62.9	105
*plc-1(tm753)*; *plc-4(jw1)*	28.3	103
*plc-1(tm753)*; *plc-2(ok1761)*	36.9	320
*plc-1(tm753)*; *plc-2(RNAi)*	31.9	354
*plc-1(tm753)*; *plc-4(RNAi)*	27.2	283
*plc-1(tm753)*; *egl-8(RNAi)*	55.7	348
*plc-1(tm753)*; *cat(RNAi)*	32.4	324

a Extrachromosomal array containing the whole genomic region including putative promoter.

b *plc-3(tm1340) is balanced over mIn1[dpy-10(e128) mIs14]* in these strains.

### Embryonic Arrest Is Observed in *plc-1* Null and Hypomorph Animals

To test *plc-1* function, we used RNAi and two alleles containing deletions: *plc-1(tm738)* and *plc-1(tm753)*. Ablation of *plc-1* function results in severely reduced zygote production due to defects in ovulation [23]. *plc-1(RNAi)*, *plc-1(tm753)* and *plc-1*(*tm738*) gave mean brood sizes of 5.8±0.9, 19.0±2.9 and 40.4±2.7 (±SEM) (N = 110, 154 and 364) respectively. Amongst the offspring produced, we observed substantial embryonic lethality (Figure 1, Table 1). The embryonic lethality observed in *plc-1(tm753)* is rescued when the *plc-1* gene is reintroduced into the *plc-1(tm753)* mutant (Table 1), thus the embryonic phenotype in these mutants is due to *plc-1* deficiency. The brood size of *plc-1(tm738)* is similar to those reported for the putative null alleles *plc-1(rx1)* and *plc-1(rx2)* [23]. Molecular analysis suggests that *plc-1(tm738)* is a null allele (Figure S1). In contrast, both embryonic survival and brood size are less affected in *plc-1(tm753)*, suggesting that it may be a hypomorph. This is confirmed by molecular analysis (Figure S1).

To test for a maternal requirement for *plc-1*, we quantified embryonic lethality in the offspring of *plc-1(tm738)*/+ and *plc-1(tm753)*/*+* heterozygotes. In both strains, the level of embryonic lethality was not significantly higher than that of wild type worms (Table 1). This suggests that, like *itr-1* [9], *plc-1* has a maternal effect. We also showed that paternal *plc-1* is not adequate to rescue embryonic lethality (data not shown).

Thus, disruption of *plc-1* function results in substantial defects in embryogenesis.

### Epidermal Cells Fail To Migrate Correctly during Morphogenesis in *plc-1* Mutants

**Figure 2:**
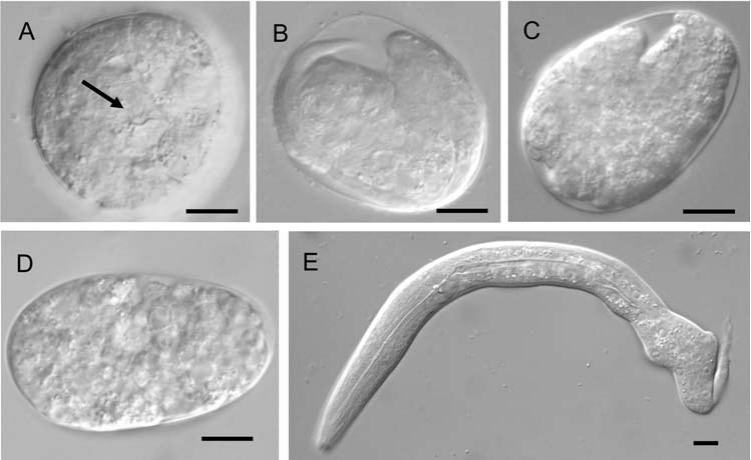
*plc-1(tm753)* embryos arrest after gastrulation and give rise to larvae with morphological defects. (A–D) DIC images showing representative examples of arrested embryos. (A) Arrested embryo in which the pharynx has developed to some extent, as the posterior bulb can be seen (arrow). (B, C) In some embryos, some morphogenetic steps have been performed. (D) Some embryos are very disrupted but have signs of cell differentiation and tissue formation. (E) Embryos that successfully hatch often produce larvae with defective body shapes. All scale bars represent 10 µm.

Ablation of *plc-1* activity could result in disruption of a number of processes required for embryonic development. Perturbing IP_3_ signalling has been shown to result in defects in cell differentiation, gastrulation and morphogenesis, as well as low penetrance defects in early cytokinesis [9],[15]. We therefore analysed the nature of the defect in *plc-1* embryos in more detail. First we noted that, in addition to arrested embryos, we also observed arrested larvae with a lumpy appearance in both *plc-1* mutants; *tm738* and *tm753* (Figure 2). Secondly, analysis of arrested embryos in *plc-1* mutants revealed that 96% of dead embryos show clear signs of cell differentiation and organ formation (see below). Thus, we did not observe any significant level of arrest prior to morphogenesis. We also observed that *plc-1* mutants lay many round and misshapen embryos (Figure 2A). However, this phenotype is not correlated with lethality, as only 64% and 36% of misshapen embryos, from *plc-1(tm738)* and *plc-1(tm753)* respectively, were arrested (N = 36 and 28 respectively).

**Figure 3:**
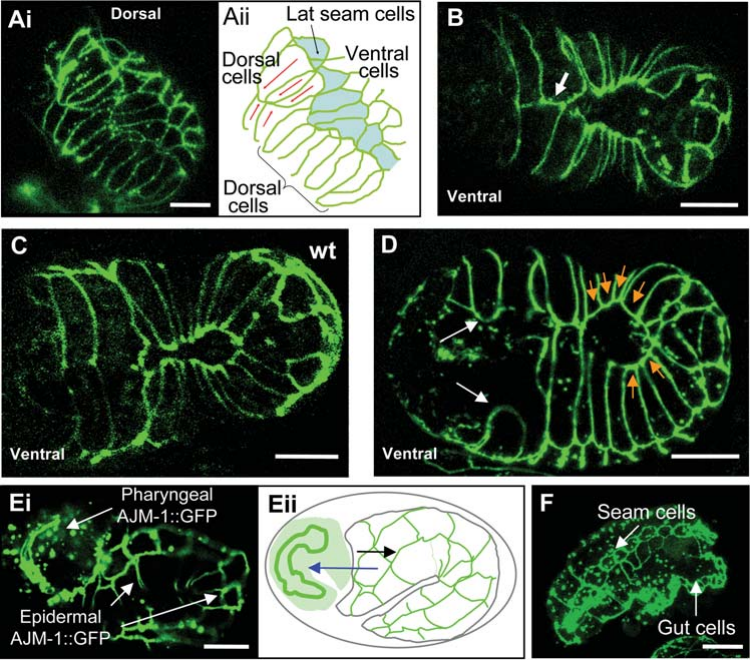
*plc-1(tm753)* embryos show defective epidermal cell migration. AJM-1::GFP was used to observe epidermal cell adherens junctions. Representative embryos are shown. (A) A *plc-1(tm753)* embryo performing morphogenesis. Ai is an image of the dorsal side of the embryo. Aii is a diagrammatic representation of this image. It is noticeable that the anterior dorsal epidermal cells have not intercalated (red arrows in Aii). (B) *plc-1(tm753)* embryo showing asymmetric migration of ventral epidermal cells during morphogenesis. Where the opposing cells have met an ectopic has been formed as one cell has made contact with both its counterpart and an adjacent cell (white arrow). A wild type embryo is shown for comparison in (C). (D) A ventral view of a *plc-1(tm753)* embryo performing ventral closure. It is apparent that the leading cells have halted during migration (white arrows). Also, the ventral cells show premature accumulation of AJM-1::GFP molecules (orange arrows). (E–F) Examples of *plc-1(tm753)* embryos showing epidermal rupture and extrusion of the interior tissues. (E) An image (Ei) and diagrammatic representation (Eii) of an embryo in which the anterior part of the epidermis is ruptured during the first steps of elongation, resulting in the pharyngeal and intestinal cells becoming external. (F) An embryo with Gex (gut on exterior) phenotype following posterior rupture. Scale bars: 10 µm.

The structure of the arrested embryos and presence of lumpy larvae suggested that *plc-1* animals might have defects in morphogenesis. To define the nature of the defects in development in more detail, we used the epithelial cell marker *ajm-1::gfp* [26] to study both arrested (i.e. terminally developed) and developing *plc-1(tm753)* embryos. Early cell divisions were normal (data not shown). All of the arrested embryos observed successfully completed gastrulation as judged by DIC microscopy (N = 186). In addition, the precursors of the gut cells showed characteristic auto-fluorescence granules (data not shown) indicating successful differentiation of intestinal cells, and most of the arrested embryos twitched vigorously, demonstrating that functional body wall muscle precursors were formed. In both arrested and developing embryos, AJM-1::GFP molecules accumulated in the apical junction domains of epidermal, intestinal and pharyngeal cells, suggesting that epithelial polarisation occurred normally in developing embryos (Figure 3). Although the embryos clearly show substantial cell differentiation, as discussed below, many embryos were highly disorganised.

**Figure 4:**
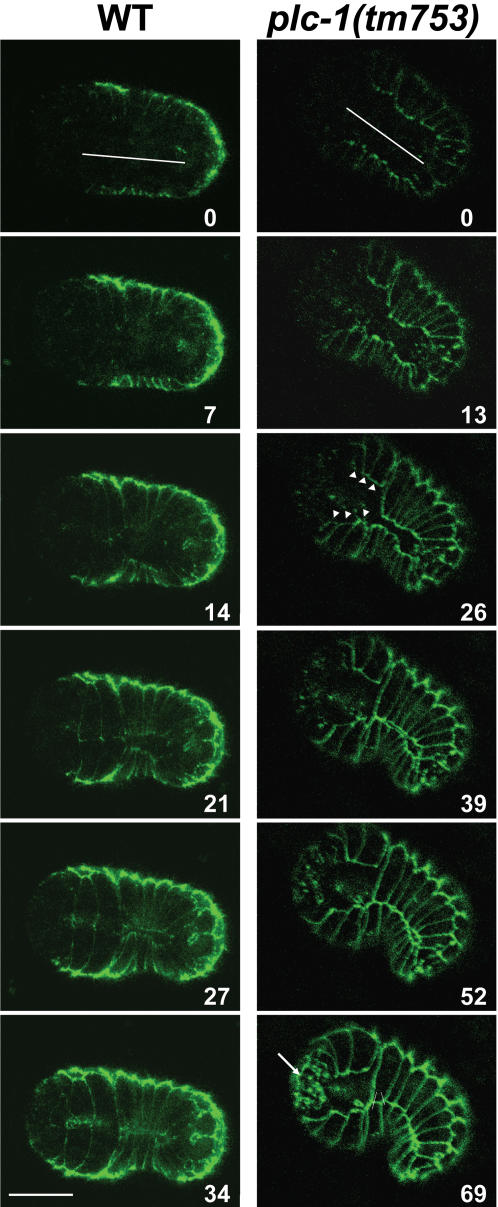
The epidermal cells of *plc-1(tm753)* embryos are defective in migration. Time lapse confocal microscopy was used to follow the migration of epidermal cells during ventral enclosure in *plc-1(tm753)* and wild type embryos expressing AJM-1::GFP. Selected time points from a representative embryo of each genotype are shown. The time in minutes is shown at the bottom right of each image. In the *plc-1(tm753)* embryo the leading cells of the mutant embryo fail to reach the midline and their opposing cell. As a result of this, the anterior part of the embryo is not enclosed by the epidermis (arrow). In addition some cells that migrate and locate their opposing counterpart also produce ectopic contacts (vertical lines). This whole process is slower in *plc-1(tm753)* embryos. Scale bar represents 10 µm.

Next, we observed the structure and behaviour of the epidermal cells that occur during morphogenesis. *plc-1(tm753)* animals have defects in both dorsal intercalation and ventral enclosure, although the latter predominate. We observed individual embryos observed under the fluorescence microscope. Approximately 11.5% showed defects in dorsal intercalation (see Figure 3A for an example). In other embryos, in which dorsal intercalation occurred successfully, ventral enclosure was disrupted (Figures 3B and D). In more extreme cases, we observed that the epidermal cells failed to form a complete sheet exhibiting gaps along the ventral mid-line. We noted that the leading cells often failed to meet, although more posterior cells were able to contact their partners (Figures 3D). The failures in ventral enclosure could reflect defects in cell migration or retraction of cells following failed junction formation. To address this we used time lapse confocal microscopy. We found that 35% of recorded embryos show defective epidermal migration (N = 23). We observed that in 50% of defective embryos the leading cells failed to migrate although more posterior cells successfully migrated and sealed (Figure 4 and Movie S1), whereas in the remaining embryos both leading and posterior cells failed to migrate. Migration of the ventral cells in *plc-1(tm753)* embryos was around 50% slower than wild type control animals even in those animals which successfully completed ventral enclosure. We also observed that cells often made both the proper contact with their opposing partner, and an ectopic contact with an additional opposing cell (Figures 3B, 3D and 4). Finally, we noted that those cells which failed to contact their partners prematurely accumulated AJM-1::GFP at the migrating edge (Figure 3D). The defects we observed are very similar to those observed in *itr-1(jc5)* embryos [9].

Presumably, as a consequence of these defects, terminally developed *plc-1(tm753)* embryos show arrest at varying points following epidermal cell movements and present a highly disorganised morphology (Figure 2). 86% of arrested embryos have tissues that are normally internal (gut, pharyngeal and other cells) externally placed (N = 186). Figures 3E and 3F show developing embryos presenting extrusion of internal tissues, just before becoming highly disorganised. Of these embryos, many show epidermal rupture in the anterior part, in agreement with the observed failure of the leading cells to complete enclosure. The remaining 10% of arrested, but clearly differentiated, embryos elongated to some extent but showed aberrant morphology.

Thus, the majority of embryonic lethality in *plc-1* mutants is caused by defects in epidermal morphogenesis. The defects observed in *plc-1* mutants are similar to the morphogenetic defects exhibited by *itr-1* mutant embryos. *itr-1(jc5)* embryos show defects including misdirected migration and premature termination of migration of the epidermal cells. In the case of *itr-1* mutants, epidermal cells have disorganised F-actin filaments and reduced filopodial protrusive activity, suggesting that ITR-1 and calcium may be regulating the cytoskeleton [9].

### PLC-1 Regulates Morphogenesis and Ovulation through ITR-1

**Figure 5:**
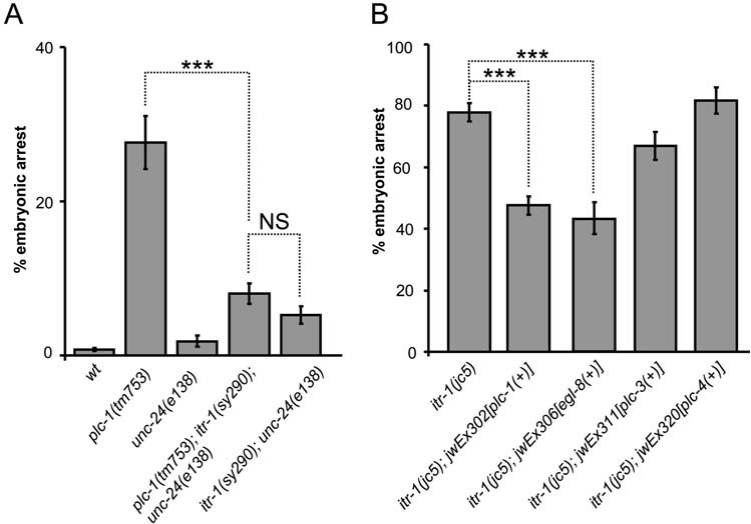
*plc-1* acts through *itr-1* to regulate embryonic development. (A) A gain-of-function allele of *itr-1*, *sy290*, is epistatic over the partial loss-of-function *plc-1(tm753)* allele, rescuing the embryonic lethality to the level of the *itr-1(sy290)* background. (B) Overexpression of *plc* genes in *itr-1(jc5)* shows that *plc-1* and *egl-8* are able to partially rescue the embryonic lethality caused by *itr-1(jc5)*. *plc-3* overexpression also slightly reduces the embryonic arrest, but is not statistically significant (p = 0.053). *plc-4* was used as a negative control. In both panels, the bars show the average lethality from offspring of 10–12 individuals and error bars represent SEMs. The two-tailed p value was determined using an unpaired t-test comparing the means of the populations. (*** p<0.0001; NS, not significant).

The epidermal defects in *plc-1* mutants resemble those in *itr-1* mutants, suggesting that PLC-1 may act through IP_3_. To assess if the *C. elegans* IP_3_ receptor, ITR-1, is a direct effector of PLC-1, we tested the effect of combining *plc-1* and *itr-1* mutants. First, we tested the effect of an *itr-1* gain-of-function allele, *itr-1*(*sy290*), on the *plc-1(tm753)* allele. *itr-1(sy290gf)*; *plc-1(tm753)* double mutants have significantly decreased embryonic arrest, compared with the *plc-1(tm753)* control animals (8%±1.3 and 27.6%±3.4 (±SEM), respectively) (Figure 5A). This strongly suggests that PLC-1 signals through ITR-1 during *C. elegans* embryonic development. Attempts to make the *plc-1*; *itr-1* loss-of-function homozygous double mutants, *plc-1(tm753)*; *itr-1(sa73ts)* and *plc-1(tm753)*; *itr-1(jc5cs)* were unsuccessful, suggesting that *plc-1*; *itr-1(l.o.f)* double mutants are not viable.

If PLC-1 signals upstream of ITR-1 during embryonic development, we hypothesised that increases in PLC-1 function, in an *itr-1(jc5)* background, should alleviate the mutant phenotype of the resulting embryos, at the restrictive temperature of 15°C. To test this hypothesis, we overexpressed *plc-1* by making strains with extrachromosomal arrays carrying the *plc-1* gene, *jwEx302[plc-1(+)]* in *itr-1(jc5)*. *jwEx302[plc-1(+)]* was able to partially rescue embryonic arrest of *itr-1(jc5)* mutants incubated at 15°C (*itr-1*(*jc5*); *jwEx302[plc-1(+)]* 47.6%±3 vs *itr-1(jc5)* 77.9%±3 (±SEM)) (Figure 5B). In addition, PLC-1 overexpression improved larval survival, as 35% (N = 468) of surviving larvae developed into fertile adults, compared to only 12% (N = 325) in *itr-1(jc5)* controls.

PLC-1 may be acting within the epidermal cells or in the underlying neuroblasts to control ventral enclosure [7]. ITR-1 is expressed in the epidermal cells [9]. *In situ* hybridisation shows that *plc-1* is widely expressed in embryos (Y Kohara personal communication). To assess the potential site of action of *plc-1* we expressed the *plc-1* cDNA using an epidermal promoter, *pelt-1* and a neuronal promoter, *punc-119.* Expression of *plc-1* from *pelt-1* partially rescued the embryonic lethality of *plc-1(tm753)* animals (14.4±3.5% vs *plc-1(tm753)* 35.6±4.6% p<0.005) suggesting that *plc-1* may be acting in the epidermal cells. We saw no change in lethality on expression of *plc-1* from the *unc-119* promoter (36.6±1.4 vs *plc-1(tm753)* 35.6±4.6%) but this could be due to a number of reasons.

These experiments strongly support the model in which PLC-1 operates through ITR-1 to signal during embryogenesis. Thus, we propose that PLC-1 is an important source of IP_3_ during epidermal cell migration and that this IP_3_ acts through ITR-1 to regulate calcium signals and the cytoskeleton, as proposed by Thomas-Virnig and co-workers [9].

We also tested whether signalling from PLC-1 by IP_3_ was used elsewhere in the animal. *plc-1* and *itr-1* are both required for ovulation [22],[23],[27],[28]. However, previous attempts to test whether *plc-1* acted through *itr-1* in the spermatheca were inconclusive because only putative null alleles of *plc-1* were available at the time [23]. We therefore measured brood size in the *itr-1(sy290gf)*; *plc-1(tm753)* animals. *itr-1(sy290)* is epistatic to *plc-1(tm753)*, increasing the average brood size in double mutants close to the level of the *itr-1(sy290)* animals (brood sizes are 64.0±7.0 and 74.7±6.6, respectively; p = 0.28) (Table 2), showing that ITR-1 works downstream of PLC-1 during both morphogenesis and strongly suggesting that this is also the case in ovulation.

**Table 2:** *itr-1(sy290gf)* rescues *plc-1(tm753)* ovulation defects.

Strain	Mean brood size	SEM	p^a^
*plc-1(tm753)*	35	0.9	0.0012
*plc-1(tm753)*; *itr-1(sy290gf)*; *unc-24(e138)*	64	7.0	
*itr-1(sy290gf)*; *unc-24(e138)*	74.7	6.6	0.2807
*unc-24(e138)*	200.6	18.6	
Wild type	237.2	5.3	

a The two-tailed p value was determined using an unpaired t-test comparing the mean brood size of the test strain to that of *plc-1(tm753)*; *itr-1(sy290gf)*; *unc-24(e138)*.

### *plc-3* and *egl-8* Can Compensate for *plc-1* and *itr-1* Loss of Function

*itr-1(jc5)* has 80–95% ([9] and Figure 5) embryonic arrest at its restrictive temperature. 30% of the embryos arrest due to defective morphogenesis (11% during ventral closure and 19% during elongation) [9]. The remaining 65% largely arrest due to earlier defects. Thus, 86% of embryos which, reach the point at which epidermal morphogenesis begins, fail to progress as a result of defects in this process. In contrast, in *plc-1* animals, we did not observe arrest prior to morphogenesis and the proportion of arrested embryos was 48% and 52% in the putative null *plc-1(tm738)* allele and in *plc-1*(*RNAi)* animals, respectively. The penetrance of the defect in *plc-1* mutants therefore appears lower than that reported for *itr-1(jc5)*, suggesting that other PLCs may be activating ITR-1. We therefore tested whether ablation of any other PLC genes could enhance the effect of *plc-1* deficiency. First, we performed RNAi against the PLCs in a *plc-1(tm753)* background. RNAi of *egl-8* resulted in a substantial increase in the levels of arrest, from 33 to 56% (Table 1). Thus *egl-8* may be acting to suppress the effects of *plc-1* depletion. RNAi of *plc-2* and *plc-4* had no effect on the level of arrest. RNAi of *plc-3* in *plc-1(tm753)* animals produced complete sterility. However, as noted previously, *plc-3(tm1340)* homozygotes rescued with extrachromosomal copies of the *plc-*3 gene do show some extra embryonic lethality (Table 1), suggesting that *plc-3* could also have a role in embryonic development.

To further analyse redundancy between the PLCs, we produced double mutants of *plc-1(tm753)* with loss-of-function alleles of *egl-8*, *plc-3*, *plc-2* and *plc-4*. Interestingly, double mutants of *plc-1* with *plc-3* (in heterozygosis) or *egl-8* produced a significant increase in arrested embryos (52.3% and 62.9% respectively, compared to 32.8% of *plc-1(tm753)* alone) (Table 1). We analysed the nature of the defects in the arrested embryos, from these double mutants, using DIC microscopy. We observed that 91.5% of *plc-1(tm753); plc-3(tm1340)*/+ and 90% of *plc-1(tm753); egl-8(n488)* arrested embryos arrest with defects in morphogenesis (N = 129 and 120 respectively). We also produced a double homozygous mutant strain of *plc-1* and *plc-3*, rescued for fertility with an extrachromosomal array carrying *plc-3*. These animals also showed increased lethality (43.5%) compared to *plc-1* alone (Table 1). Thus, reduction of *plc-3* or *egl-8* function enhances the phenotype of *plc-1*, suggesting that PLC-3 and EGL-8 are able to function redundantly with PLC-1. Analysis of animals carrying rescuing transgenes containing *plc-3* fused to GFP revealed that *plc-3* is expressed in a range of embryonic cells including the epidermal cells during morphogenesis (data not shown). Thus *plc-3* may contribute to successful morphogensis in normal circumstances although to dissect this would require either a *plc-3* hypomorph, which is not currently available, or that the *plc-3* ovulation defect is specifically rescued, allowing us to analyse embryos. *egl-8* alone does not give rise to embryonic lethality, suggesting that either another PLC (perhaps *plc-1*) is able to completely compensate for loss of *egl-8*, or that *egl-8* does not normally have a role in epidermal cell movements, but is able to compensate for loss of *plc-1* in mutants. Compatible with the latter explanation we were unable to detect any expression of rescuing *egl-8::gfp* fusions during early or mid-stage embryogenesis.

We also tested whether any of the other PLCs could act upstream of *itr-1* and improve survival. We induced excess PLC function, in an *itr-1* loss-of-function environment, by producing strains carrying extrachromosomal arrays of *egl-8* (*jwEx306[egl-8(+)]*), *plc-3* (*jwEx311[plc-3(+)]*) and *plc-4* (*jwEx320[plc-4(+)]*), in *itr-1(jc5)*. *jwEx306[egl-8(+)]* gave a substantial and significant improvement in survival, whilst *jwEx311 [plc-3(+)]* gave a small reduction in lethality which was not statistically significant (Figure 5). These results are compatible with those obtained by testing the role of the other PLCs in *plc-1(tm753)* animals and again suggest that EGL-8/PLC-β is either involved in epidermal cell movements or is able to act in this pathway in certain artificial situations.

We conclude that *egl-8* and *plc-3* may play a role in morphogenesis and/or may be able to augment *plc-1* activity. No function in cell migration has, to our knowledge, been described for PLC-β. On the other hand, PLC-γ1 has been shown to play a role in epithelial cell migration in mammals where it interacts directly with villin [29],[30]. If this relationship is conserved in *C. elegans*, this may provide a mechanism of action of PLC-3 in the epidermal cells during cell migration.

### Phospholipase C-ε Regulates Embryonic Morphogenesis in *C. elegans*

We conclude that PLC-1, the *C. elegans* orthologue of PLC-ε, has an important role in the epidermal cell movements that underlie morphogenesis in the *C. elegans* embryo. This is the first report of a role for PLC-ε in morphogenesis, although previous reports have suggested roles in the development of mouse heart valves [31], and human glomeruli [32]. The results of our analyses demonstrate that the PLC-1 signal is transduced through ITR-1. Previous work implicates *itr-1* in the regulation of Ca^2+^ signals that in turn control cytoskeletal activity in the migrating epithelial cells [9]. The discovery of PLC-ε as a component of this process suggests a mechanism by which IP_3_ and calcium signalling may be linked to signalling through small GTPases of the Rho and Ras family, which are known to play important roles in these same events.

Our results identify a new component of the molecular mechanism that controls epidermal cell movements. This mechanism may be important in cell movements in morphogenesis in other animals and in related processes such as wound healing.

## Materials and Methods

### Strains and Worm Culture

Worms were cultured using standard techniques and media [33]. Strains used in this work and their origins are listed in Table S1. The strain carrying the allele *plc-4(jw1)* is a complete deletion of the *plc-4* gene, and was generated by gene targeting and homologous recombination (unpublished results), following a modified version of a previously described method [34]. All strains were maintained at 20°C, unless otherwise stated.

### RNA Interference (RNAi)

RNAi, by feeding of the PLC genes, was carried out using Escherichia coli HT115 carrying derivatives of the vector pPD129.36 [35], containing ∼1 kb of the cDNA of each PLC gene [21]. As a control, we used a derivative of pPD129.36 with the chloramphenicol acetyl transferase (cat) gene from E. coli. Several L3 hermaphrodites were placed onto each feeding plate and incubated at 20°C for 24 hours and then transferred every 24 hours onto fresh separate feeding plates. Phenotypes were scored as described below.

### Analysis of *plc-1* cDNA from *plc-1(tm753)* Worms

Total RNA was extracted from worms using a previously described method [14]. cDNA was produced using Superscript III (Invitrogen), following the manufacturer's instructions. *plc-1* cDNA was amplified using two rounds of PCR. The primers RV1157 (CAG CAA ATA GCC TGG AGA GT) and RV1159 (AAC GAG CAC TGA GAA TGC CA) were used in the first round. The second round PCR used RV1158 (CAC AAT CTC GTG TGA TTC CA) and RV1160 (GGC GGA CCA GAT TGT GAC GA).

### Brood Size Assay

Brood size was measured by placing L4 larvae onto individual plates, and then transferring them every 12–24 hours, at 20°C, for as long as the animals laid embryos. The number of progeny on each plate was counted ≥24 hours after removing the parent. We define brood size as the total number of embryos produced, regardless of whether these embryos hatch or not.

### Embryonic Lethality

To investigate embryonic lethality, we performed brood assays as stated above, and determined the number of arrested embryos and larvae. We used 10 to 12 parental animals per strain. We considered embryos as arrested when they failed to hatch within 24 hours after removal of the parent. In the case of strains containing the cold-sensitive allele *itr-1(jc5)*, when incubated at 15°C, embryos were allowed 36 hours in which to hatch. Experiments were performed at least three times and representative experiments are shown.

### Examination of Embryonic Development

Embryos were isolated either by dissecting from adults or by bleaching of worm populations. For routine examination embryos were mounted in embryo culture medium [36]. Embryos were then analysed as they developed or left for 18 hours at 20°C to develop for terminal phenotyping. DIC microscopy was performed using a Zeiss Axioskop 2 microscope (Zeiss, Göttingen, Germany), equipped with a Q imaging, Micro Publisher 5.0 RTV digital camera (Burnaby, ON, Canada). Fluorescence microscopy was performed using a Leica SP1 confocal microscope (Wetzlar, Germany).

Embryos for time lapse (4D) confocal microscopy were mounted in embryo culture medium [36]. Time lapse microscopy was performed using a Leica SP5 confocal microscope, and 3D images were collected every 2–4 minutes.

### Construction of Transgenic Strains

Standard molecular biology techniques were used to produce DNA constructs [37]. For PLC overexpression experiments, 10 ng/µl of the PLC-construct DNA was injected into worms, together with pRF4 (a plasmid containing a dominant *rol-6* marker), at a final total DNA concentration of 100–150 ng/µl, using previously described methods [38].

To make strains carrying arrays containing the *plc-1* gene, e.g *jwEx302[plc-1(+)],* the fosmid WR64BC06 was injected. WR64BC06 contains the whole *plc-1* gene including the putative promoter, and was obtained from Geneservice (Cambridge, UK). *plc-3 arrays, e.g. jwEx311[plc-3(+)]*, contain the plasmid pOB115, which has the putative promoter (2.8 kb upstream of the ATG start site) and *plc-3* gene in frame with *gfp*. To obtain the plasmid pOB115, we amplified the coding region of *plc-3* (6186 bp) using the following primers: OB1279 (CGA TGG CGC GCC ATG CAA CAC GGC TCA CTT GG) and OB1281 (ATC GGC GGC CGC TGA TTT ACT ACT TTT TCC AAA TGA GAA C). This fragment was cloned in frame to YFP using the sites AscI and NotI, into the plasmid pSP002 (S. Parker and H. Baylis, unpublished). Then, the putative promoter of *plc-3* was amplified using OB1278 (CGA TCT GCA GGC CAC GTG TCT CGA TAG AAT G) and OB1280 (ATC GGG CGC GCC GCT GAG AAA TTG AAG GAT TTA TGA AAT TGG) and cloned before the coding region using PstI and AscI. *plc-4* arrays, e.g. *jwEx320[plc-4(+)]*, include pRV011 which contains the complete *plc-4* gene and its putative promoter (a region spanning from 0.6 kb upstream of the ATG start site and 2.2 kb downstream of the stop codon). To obtain pRV011 we amplified the *plc-4* loci (5853 bp) using the primers RV760 (GGA ACC GCG GCC AAT CAA TAC TTC CAT TGC C) and HAB596 (ATG CGG CCG CCC ATT TCT CGG TCA AAG TGA TTC C) and cloned into pGEM-T (Promega). *jwEx306[egl-8(+)]* contains the plasmid KP#440 (A gift from S Nurrish), which consists of a minigene of *egl-8* containing its promoter and first six exons fused to exons 7–20 from cDNA.

To test the site of action of PLC-1 we used arrays expressing *plc-1* from epidermal and neuronal promoters. *jwEx325* contains pAN51. pAN51 was constructed by placing the cDNA of *plc-1* under the control of the *elt-1* promoter. It also contains an “operonic” GFP downstream of the *plc-1* cDNA which expresses GFP, but not fused to PLC-1, from the same promoter. To generate this plasmid we used the Gateway technology (Invitrogen). To obtain the *elt-1* promoter, we used as primers AN2297 (GGG GAC AAG TTT GTA CAA AAA AGC AGG CTG TAG ACG GTT GCC GTT TGA ATT TC) and AN2298 (GGG GAC AAC TTT GTA TAG AAA AGT TGG GTG ATC ATG GTC CTC GCC ACC GAC). The array *jwEx333* contains pAN53. pAN53 expresses the cDNA of *plc-1* under the control of the *unc-119* promoter, and was constructed in a similar way as pAN51. To obtain the *unc-119* promoter we used as a primers AN2142 (GGG GAC AAG TTT GTA CAA AAA AGC AGG CTG TAA GCT TCA GTA AAA GAA GTA G) and AN2277 (GGG GAC AAC TTT GTA TAG AAA AGT TGG GTG ATA TAT GCT GTT GTA).

### Statistical Analyses

Data are presented as means±SEM. Statistical significance was determined using Student's two-tailed t-test, using the online resources of Graphpad (http://www.graphpad.com). P values are shown to indicate statistical significance.

## Supporting Information

Figure S1 Molecular lesions in the *plc-1(tm738)* and *plc-1(tm753)* alleles. (A) The genomic organisation of the *plc-1* gene showing exons (blue boxes) and introns (black lines). The extent of the deletions in *tm753* and *tm738* are shown in red. *tm738* is a small deletion but produces a change of frame which is likely to result in a severely truncated protein and is likely to be a null allele. *tm753* removes exon 6 and part of 7. Nevertheless, a cDNA clone obtained by RT-PCR in this strain contained an in-frame messenger, which is predicted to produce a version of the protein with an internal deletion. The structure of this mRNA is shown at the bottom of the panel, together with the sequence of the cryptic splice site used. (B) The protein module structure of PLC-1, showing the effects of the two deletions. *tm753* results in a protein with a deletion that covers parts of the CDC-25 and PH domains. The blue dashed line under *tm738* represents out of frame peptide sequence. (C) The region in which the *plc-1(tm753)* allele lies is highly conserved between mammals and nematodes. Amino acid residues in red indicate the deleted sequence in the putative *tm753* peptide.
(1.51 MB TIF)

Table S1 Strains used in this work.
(0.07 MB DOC)

Movie S1 The epidermal cells of *plc-1(tm753)* embryos are defective in migration. Time lapse confocal microscopy was used to follow the migration of epidermal cells during ventral enclosure in *plc-1(tm753)* embryos expressing AJM-1::GFP. In this embryo the leading cells fail to reach the midline and their opposing cell. As a result of this, the anterior part of the embryo is not enclosed by the epidermis. This whole process is slower in *plc-1(tm753)* than wild type embryos.
(1.06 MB CDR)
