# Supplementary figures and images for: Phospholipase C-ε Regulates Epidermal Morphogenesis in Caenorhabditis elegans
Source: PLoS Genet. 2008 Mar 28;4(3):e1000043. doi: 10.1371/journal.pgen.1000043 (PMC2274882; doi:10.1371/journal.pgen.1000043)

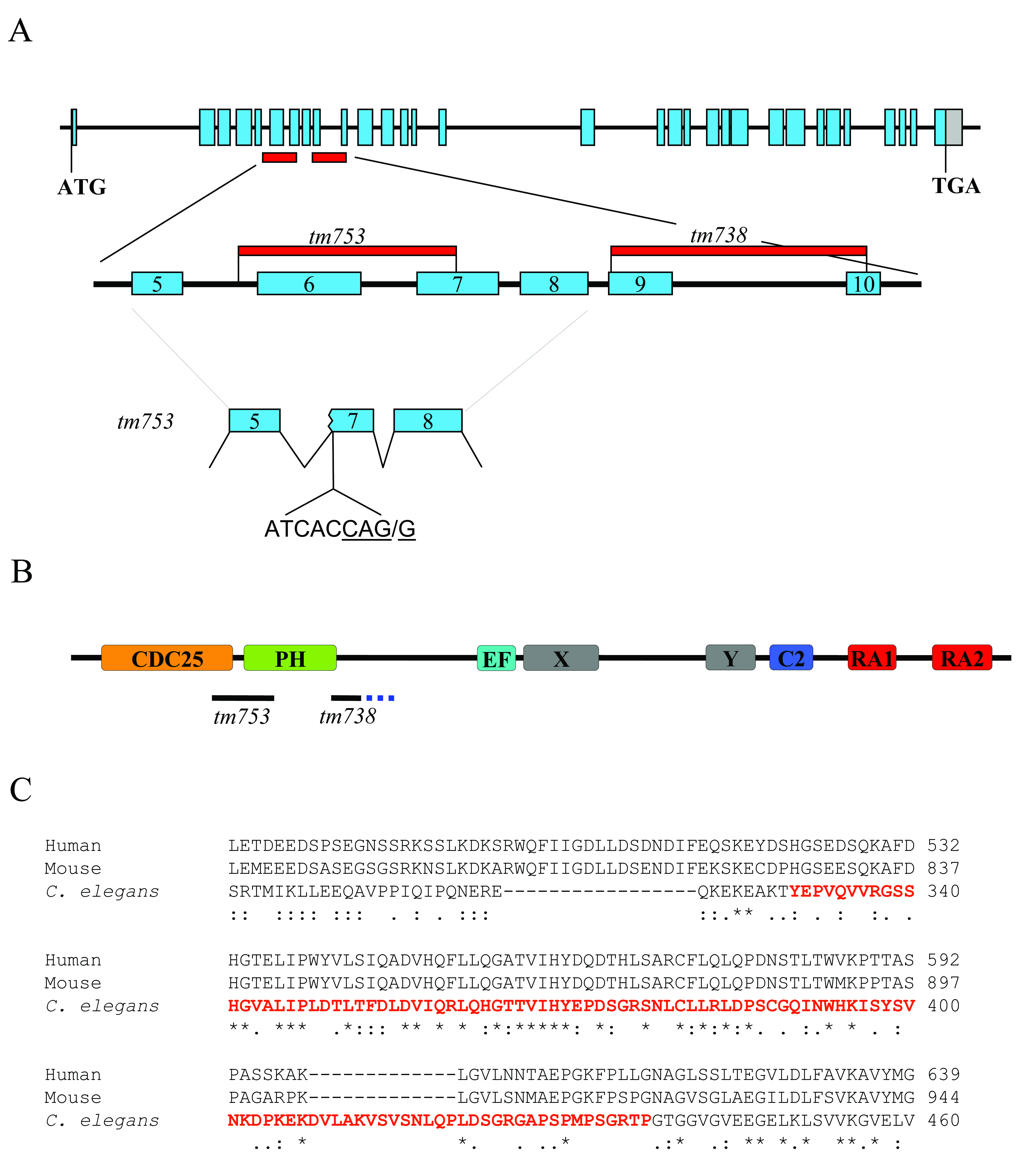

Supplement: Figure S1 — Molecular lesions in the plc-1(tm738) and plc-1(tm753) alleles. (A) The genomic organisation of the plc-1 gene showing exons (blue boxes) and introns (black lines). The extent of the deletions in tm753 and tm738 are shown in red. tm738 is a small deletion but produces a change of frame which is likely to result in a severely truncated protein and is likely to be a null allele. tm753 removes exon 6 and part of 7. Nevertheless, a cDNA clone obtained by RT-PCR in this strain contained an in-frame messenger, which is predicted to produce a version of the protein with an internal deletion. The structure of this mRNA is shown at the bottom of the panel, together with the sequence of the cryptic splice site used. (B) The protein module structure of PLC-1, showing the effects of the two deletions. tm753 results in a protein with a deletion that covers parts of the CDC-25 and PH domains. The blue dashed line under tm738 represents out of frame peptide sequence. (C) The region in which the plc-1(tm753) allele lies is highly conserved between mammals and nematodes. Amino acid residues in red indicate the deleted sequence in the putative tm753 peptide. (1.51 MB TIF) [file pgen.1000043.s001.tif]
