# Supplementary material for: Phospholipase C-ε Regulates Epidermal Morphogenesis in Caenorhabditis elegans
Source: PLoS Genet. 2008 Mar 28;4(3):e1000043. doi: 10.1371/journal.pgen.1000043 (PMC2274882; doi:10.1371/journal.pgen.1000043)
Supplement: Table S1 — Strains used in this work. (0.07 MB DOC) [file pgen.1000043.s002.doc]

| **Table S1.** **Strains used in this work** | | |
| --- | --- | --- |
| **Strain** | **Genotype^a^** | **Reference** |
| Bristol N2 | wild type | [1] |
| *itr-1(jc5)* | *itr-1(jc5)*IV | [2] |
| JT73 | *itr-1(sa73)*IV | [3] |
| CB138 | *unc-24(e138)*IV | [1] |
| PS2582 | *itr-1(sy290)*; *unc-24(e138)*IV | [4] |
| SU93 | *jcIs1* IV | [5] |
| DR2078 | *mIn1*[*dpy-10(e128)* *mIs14*]II/*bli-2(e768)*II *unc-4(e120)*II | [6] |
| HB372 | *plc-1(tm753)*X | This work |
| HB389 | *plc-1(tm738)*X | This work |
| HB679 | *plc-2(ok1761)*V | This work |
| HB652 | *plc-3(tm1340)*II/*mIn1*[*dpy-10(e128)mIs14*]II | This work |
| HB653 | *plc-3(tm1340)*II; *jwEx311[plc-3(+)]* | This work |
| HB349 | *plc-4(jw1)*IV | RVM unpublished |
| MT1083 | *egl-8(n488)*V | [7] |
| HB391 | *plc-1(tm753)*X; *plc-2(ok1761)*V | This work |
| HB392 | *plc-1(tm753)*X; *plc-3(tm1340)*II/*mIn1*[*dpy-10(e128)mIs14)*II | This work |
| HB393 | *plc-1*(*tm753*)X; *plc-3*(*tm1340*)II; *jwEx311[plc-3(+)]* | This work |
| HB372 | *plc-1(tm753)*X; *plc-4(jw1)*IV | This work |
| HB394 | *plc-1(tm753)*X; *egl-8(n488)*V | This work |
| HB395 | *plc-1(tm753)*X; *jcIs1* IV | This work |
| HB396 | *plc-1(tm753)*X; *jwEx302[plc-1(+)]* | This work |
| HB397 | *itr-1(jc5)*X; *jwEx302[plc-1(+)]* | This work |
| HB398 | *itr-1(jc5)*X; *jwEx306[egl-8(+)]* | This work |
| HB701 | *itr-1(jc5)*X; *jwEx311[plc-3(+)]* | This work |
| HB700 | *itr-1(jc5)*X; *jwEx320[plc-4(+)]* | This work |
| HB380 | *plc-1(tm753)*X; *itr-1(sy290)*IV; *unc-24(e138)*IV | This work |
| ^a^ All mutant strains were outcrossed at least three times, except *plc-2(ok1761)*, which was outcrossed once. JT73, DR2078, CB138, PS2582, MT1083 and SU93 were supplied by the *Caenorhabditis* Genetics Centre (University of Minnesota, MN). *plc-1(tm753)*, *plc-1(tm738)* and *plc-3(tm1340)* were supplied by the NBRP (Tokyo Women's Medical University School of Medicine). *plc-2(ok1761)* was supplied by the *C. elegans* Gene Knockout Project (Oklahoma Medical Research Foundation). | | |
